# What is the impact of increasing the prominence of calorie labelling? A stepped wedge randomised controlled pilot trial in worksite cafeterias

**DOI:** 10.1016/j.appet.2019.05.035

**Published:** 2019-10-01

**Authors:** Milica Vasiljevic, Georgia Fuller, Mark Pilling, Gareth J. Hollands, Rachel Pechey, Susan A. Jebb, Theresa M. Marteau

**Affiliations:** a Behaviour and Health Research Unit, Institute of Public Health, University of Cambridge, Cambridge, UK; b Department of Psychology, Durham University, Durham, UK; c Nuffield Department of Primary Care Health Sciences, University of Oxford, Oxford, UK

**Keywords:** Choice architecture, Nudging, Stepped wedge trial, Randomised controlled trial, Workplace interventions, Calorie labelling

## Abstract

**Background:**

Calorie labelling may help to reduce energy consumption, but few well-controlled experimental studies have been conducted in real world settings. In a previous randomised controlled pilot trial we did not observe an effect of calorie labelling on energy purchased in worksite cafeterias. In the present study we sought to enhance the effect by making the labels more prominent, and to address the operational challenges reported previously by worksites.

**Methods:**

Three worksite cafeterias were randomised in a stepped wedge design to start the intervention at one of three fortnightly periods between March and July 2018. The intervention comprised introducing prominent calorie labelling for all cafeteria products for which calorie information was available (on average 87% of products offered across the three sites were labelled). Calorie content was displayed in bold capitalised Verdana typeface with a minimum font size of 14 *e.g.***120 CALORIES**. Feasibility and acceptability were assessed using post-intervention surveys with cafeteria patrons and semi-structured interviews with managers. Effectiveness was assessed using total daily energy (kcal) purchased from intervention items across the three sites, analysed using semi-parametric GAMLSS models.

**Results:**

Recruitment and retention of worksite cafeterias proved feasible: all three randomised sites successfully completed the study. Post-intervention feedback suggested high levels of intervention acceptability: 87% of responding patrons wanted calorie labelling to remain in place. No effect of the intervention on daily energy purchased was observed: −0.6% (95%CI -2.5 to 1.2, *p* = .487). By-site analyses showed similar null effects at each of the three sites, all *p*s > .110.

**Conclusions:**

There was no evidence that prominent calorie labelling changed daily energy purchased across three English-based worksite cafeterias. The intervention was feasible to implement and acceptable to patrons and managers.

## 1 Background

Excess energy intake and poor diet quality leading to obesity are the leading causes of the rising incidence of non-communicable diseases and excess mortality in England and worldwide (Gakidou et al., 2017; Naghavi et al., 2017; Steel et al., 2018; Swinburn et al., 2019). Interventions aimed at reducing energy intake and/or improving diet quality are therefore key to improving the health of populations (Swinburn et al., 2015). Recent evidence suggests that interventions that change aspects of the physical environment or ‘choice architecture’ may be more effective at changing dietary behaviour including reducing energy intake than more traditional interventions requiring conscious engagement, such as educational campaigns (Hollands, Marteau & Fletcher, 2016; Hollands et al., 2017; Marteau, Hollands, & Fletcher, 2012; Thaler & Sunstein, 2008).

One potential promising choice architecture intervention that alters environmental cues that are temporally and physically proximal to the point of choice is calorie labelling (Hollands et al., 2017; Zlatevska, Neumann, & Dubelaar, 2018). In the USA, calorie labelling for all food products sold in out-of-home food retail environments has been mandatory since 2010 (FDA, 2014). In England the government is considering implementing similar legislation to make calorie labelling mandatory for the out-of-home sector (Department of Health and Social Care, 2018).

Though potentially impactful and overwhelmingly desired by customers, the estimated effect size of calorie labelling on energy purchased has been found to vary across studies, with a paucity of experimental evidence, particularly in field settings amongst general population samples. The evidence from a recent Cochrane review of nutritional labelling suggests that if calorie labels were added to menus or put next to foods in restaurants, coffee shops and cafeterias this could reduce energy purchased by about 47 calories (7.8%) per meal on average (Crockett et al., 2018). The synthesised evidence was, however, derived from three studies, all conducted in the USA and assessed as being of low quality using the GRADE assessment tool due to very serious risk of bias.

Another recent systematic review synthesised evidence from 186 mainly US-based studies. These included both experimental and non-experimental studies, conducted in laboratory or field settings. This synthesis led to an estimated smaller effect of calorie labelling – amounting to a reduction of approximately 27 calories (4.6%) per meal (Zlatevska et al., 2018). This systematic review also accounted for study heterogeneity, showing that the effect size of calorie labelling was larger in laboratory (hypothetical-choice) studies, and larger amongst women and those who were overweight.

A third recent systematic review provided evidence that calorie labelling may be more effective amongst those of higher socio-economic position (SEP), though these conclusions derived from narrative and not quantitative synthesis of a small number of studies measuring the impact of calorie labelling across different SEP groups (Sarink et al., 2016). Finally, a fourth systematic review conducted by Shangguan and colleagues estimated that calorie labelling could reduce total energy intake by 5.8% per meal (Shangguan et al., 2019). In this systematic review, the impact of nutrient content labelling *vs.* calorie labelling was examined, but there was no sufficient evidence to conclude that one of these types of labelling is more effective in lowering energy intake, mainly due to the small number of studies available for these moderation analyses. In sum, these recent systematic reviews suggest that there remains considerable uncertainty about the potential impact of calorie labelling and that calorie labelling may have differential impacts amongst different groups, and may be dependent on the intervention setting.

In a recent study, we sought to build on these heterogeneous findings by examining the impact of calorie labelling upon energy purchased using an experimental design across six worksite cafeterias in England (Vasiljevic et al., 2017, 2018). We found that, although highly acceptable to cafeteria patrons and managers, the calorie labelling intervention had no effect upon energy purchased across the six sites. At one of the six sites, there was a statistically significant reduction in total calories purchased, with an estimated reduction of 6.6% [95% CI -12.9% to −0.3%], which diminished over time.

There were several possible explanations for the lack of an observed effect in five out of the six worksites in this study. The calorie labels were designed to be visible to the customer at the point of choice, and were therefore presented in the same font style and size as the product price. This design may, however, have inadvertently decreased the impact of the intervention by making the calorie information less distinguishable from the other information on the label. There were also some operational difficulties in collecting the primary outcome measure which limited the precision of the data collected in the initial trial. For example, four of the six sites recorded a small number of their food/drink items – such as sales of different carbonated drinks - under the same till button, thus preventing full disaggregation of sales of products with different energy content.

In the current replication and extension study we therefore sought to use visually-enhanced calorie labels designed to communicate more prominently the energy content. In addition, we aimed to work closely with the catering teams and others in the participating sites to improve their till systems for data capture, and accordingly, to improve the estimates of the potential impact of calorie labelling on energy purchased.

The aims of the present study are:

(1) to assess the feasibility of recruiting eligible worksites, and identify potential barriers to the feasibility and acceptability of implementing prominent calorie labelling; and
(2) to estimate the impact of prominent calorie labelling designed to clearly communicate energy content upon energy purchased in worksite cafeterias.

## 2 Methods

### 2.1 Sample

Three worksite cafeterias in England were recruited to take part in the study via a collaboration with the Institute of Grocery Distribution (IGD) (Institute of Grocery Distribution, 2018). Worksites were eligible if they were based in England, employed more than 300 employees and had the ability to provide data on daily sales of individual items and their energy content. Due to the pilot nature of the study, a sample size of three sites was selected prior to enrolment as a pragmatic number with which to address the study aims within available resources.

**Fig. 1:**
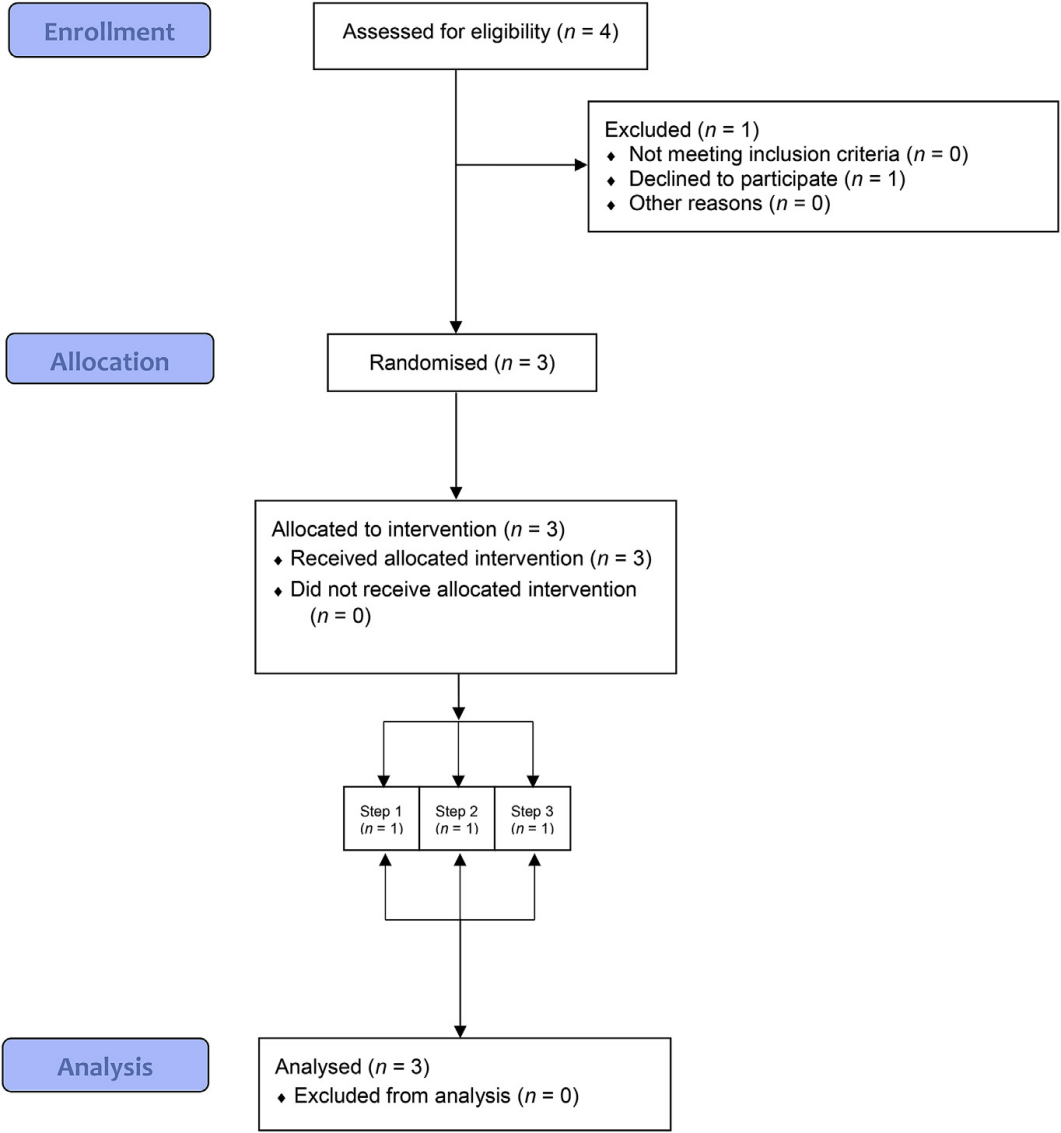
CONSORT diagram of participant flow through the study.

We approached the managers of four sites that were part of a Healthy Eating in the Workplace Advisory Group organised by IGD and had already expressed interest in participating in studies. Sites were then screened for eligibility. All four sites were deemed eligible on the criteria reported above. Of the four sites approached, three agreed to participate in this pilot study and were therefore randomised to the time at which to implement the intervention. Enrolment of sites into the study was conducted by two members of the research team (MV and GF). The flow of participating sites through the pilot trial is shown in the CONSORT diagram in Fig. 1. The demographic characteristics of employees at the three sites are summarised in Table 1 (these data were provided by the worksite Human Resource departments with all data points provided in aggregate form as they appear in the table). The baseline characteristics of intervention items across the three sites are summarised in Table 2.

**Table 1:** Staff demographic characteristics across the three sites.

Categories	Site 1 (*n* = 2205)	Site 2 (*n* = 337)	Site 3 (*n* = 405)
Employment Type (*n*/%)			
Full Time	2011 (91%)	335 (99%)	245 (60%)
Part Time	132 (6%)	2 (1%)	20 (5%)
Temporary	62 (3%)	0	140 (35%)
Gender (*n*/%)			
Male	824 (37%)	278 (82%)	243 (60%)
Female	1381 (63%)	59 (18%)	162 (40%)
Age (*n*/%)			
18–24	164 (7%)	42 (12%)	41 (10%)
25–34	884 (40%)	148 (44%)	101 (25%)
35–44	572 (26%)	96 (28%)	142 (35%)
45–54	416 (19%)	36 (11%)	73 (18%)
55–64	165 (7%)	15 (4%)	36 (9%)
65+	4 (0.2%)	0	12 (3%)
Role Type (*n*/%)			
Higher Managerial	25 (1%)	1 (0.3%)	20 (5%)
Intermediate Managerial	240 (11%)	8 (2%)	81 (20%)
Supervisory or Clerical/Junior Managerial Skilled	622 (28%)	20 (6%)	170 (42%)
Manual Worker	1125 (51%)	50 (15%)	114 (28%)
Semi or Unskilled Worker	193 (9%)	228 (68%)	20 (5%)
Other	0	11 (3%)	0

*Note.* Sites 1 and 3 did not have any staff in the 'other' category (e.g., students). Site 2 did not have any temporary employees or anyone over the age of 65 years old.

**Table 2:** Baseline sales data of intervention items across the three sites.

Categories	Site 1 (*n* = 2205)	Site 2 (*n* = 337)	Site 3 (*n* = 405)
Number of Daily Transactions [Mean (SD)]	2365.6 (222.2)	226.5 (21.2)	159.2 (24.9)
Main Meal Kcal [Mean [SD] (min, max)]	418.4 [387.3] (95, 1614)	415.0 [162.4] (154, 829)	542.0 [238.5] (144, 1025)
Drink Kcal [Mean [SD] (min, max)]	71.0 [58.9] (0, 216)	121.2 [67.0] (0, 366)	81.2 [57.5] (0, 240)
Snack Kcal [Mean [SD] (min, max)]	163.2 [166.4] (27, 657)	243.1 [126.0] (35, 770)	207.8 [107.1] (21, 576)
Mean Cost of Main Meal (£) [Mean [SD] (min, max)]	1.51 [0.89] (0.80, 3.90)	2.69 [0.67] (0.60, 3.90)	2.89 [0.53] (1.99, 3.95)

*Note.* Sales of main meals at Site 3 are recorded with side dishes as the default option. At Site 3, employees must request if they do not want a particular side to be automatically included with their main meal.

### 2.2 Design and procedure

**Fig. 2:**
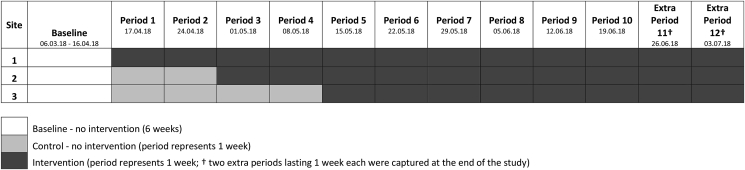
A graphical presentation of the study's stepped wedge design**.**

The study used a stepped wedge randomised controlled trial design (Brown & Lilford, 2006; Hemming, Haines, Chilton, Girling, & Lilford, 2015; Hughes, 2007). This design was chosen since it allows the intervention to be tested across all eligible sites thus maximising study power; as well as allowing a more robust control of unexpected events over time since the roll out of the intervention occurs sequentially across the different sites. Between March and July 2018 three worksite cafeterias were sequentially randomised to receive the intervention after an initial baseline period of at least six weeks (see Fig. 2). Sites were randomised to implement the intervention at one of three, two-weekly intervals. The randomisation of sites to the intervention sequence was performed by a statistician (MP) using computer-generated random numbers (the statistician was blinded to the identity of sites throughout the randomisation process). The protocol for this pilot trial was prospectively registered [ISRCTN20474205] (for more details see http://www.isrctn.com/ISRCTN20474205).

During the 6-week pre-intervention period, routine cafeteria service continued while information was collected on the energy content of food available and on the sales each day. The intervention periods were planned to be at least equal in length to the pre-intervention period – *i.e.* the third site implementing the intervention for at least six weeks – so that a best estimate of intervention impact could be obtained. Two further intervention weeks were run at the end of the trial for all three sites. Accordingly, the period of intervention lasted between eight to twelve weeks, depending on randomisation sequence within the stepped wedge design. It was not possible to blind the caterers who implemented the intervention to intervention assignment. Patrons of the cafeterias were not informed that the introduction of prominent calorie labelling was being evaluated as part of a study.

The research team trained and instructed the catering teams across the three worksites on how to implement the intervention prior to the study start date and worked closely with the catering managers during intervention implementation. Prior to the commencement of the study, till systems were discussed and all worksites were instructed to use individual till buttons for each individual product in their cafeterias. Where this was not practically possible (*e.g.,* due to a large product offering as in Site 1), a few till buttons were reprogrammed to capture a few products of the same category that were similar in energy content (with the difference in energy ranging between ±30 kcal). Compliance with intervention implementation was measured by one member of the research team who conducted fortnightly visits to the worksites and recorded any deviations from the study protocol. Sales data were collected from all three sites over the period 6th March to 9th July 2018. The catering teams provided the research team with data on the energy content of food and drink items as well as till records of the sales data for each day throughout the study period.

### 2.3 Intervention

The intervention comprised labelling all cafeteria products for which calorie information was available with their energy content *e.g.,* **120 CALORIES**. Following evaluation of the impact of the labelling intervention in our previous study (Vasiljevic et al., 2018), we aimed to enhance the presentation of calorie information by displaying this information more prominently in the current study. A literature review provided the basis for design features to make the labels more prominent.

The findings from the review suggested that typefaces such as Verdana (Chaparro, Shaikh, Chaparro, & Merkle, 2010; Franken, Podlesek, & Možina, 2015; Josephson, 2008) increased readability compared to Times New Roman and Arial, with bolded (Silver & Braun, 1993), larger fonts (Silver & Braun, 1993; Silver et al., 1994; Vigilante and Wogalter, 1999; Wogalter & Vigilante, 2003), and uppercase letters (Ohyama & Sagawa, 2016) also aiding readability. Increasing white space around a message and using high contrasts such as black text on a white background could also enhance readability (Arditi & Cho, 2007; Rousek & Hallbeck, 2011). In order to maximise effectiveness of the labelling, the extant literature suggested combining these features (Laughery, Young, Vaubel, & Brelsford, 1993; Vigilante and Wogalter, 1999). For more information on how we incorporated these findings from the extant literature in the design of the new prominent calorie labels see the Calorie Labelling Manual document in Online Supplementary Materials.

As in our previous study, the labels were designed to be visible and legible to the customer from where they would be standing at the point of choice. Labels also contained calorie information by product portion size by denoting ‘per slice’, ‘per ladle’, or ‘per average bowl/serving’. Salad bars, deli bars, hot drinks, and vending machine items were excluded from the intervention because of challenges in reliably implementing calorie labelling for these items (see the Calorie Labelling Manual document in Online Supplementary Materials for more details).

**Fig. 3:**
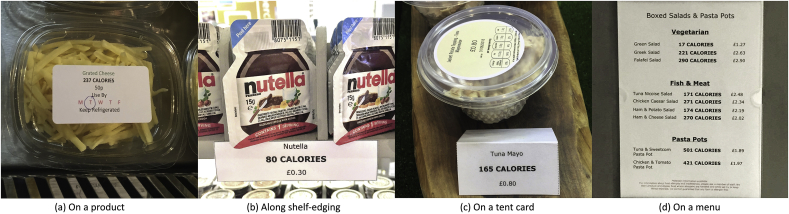
Examples of calorie labelling: a) on a product; b) along shelf-edging; c) on a tent card; and d) on a menu.
(2) Along shelf edging at point of choice (see Fig. 3b);
(3) On tent cards placed next to products (see Fig. 3c); and
(4) On menus (printed or electronic via email or screens; see Fig. 3d).

In the present study calorie information was provided in one of four different places:

(1) On products (see Fig. 3a);

### 2.4 Measures

#### 2.4.1 Feasibility and acceptability

The feasibility and acceptability of the intervention implementation in the present study were captured using the following indicators:

(1) *Feasibility of recruiting and retaining eligible worksites.* This was assessed by examining recruitment and drop-out rates;
(2) *Feasibility of implementing the assigned intervention.* This was assessed after initial visits to worksite cafeterias by the research team, in discussions and formal interviews with worksite managers and catering teams, and through examination of the sites' sales data;
(3) *Acceptability of the intervention.* This was measured via surveys distributed to cafeteria patrons, and qualitative interviews with worksite managers/caterers. In the surveys cafeteria patrons were asked: “*How did you feel about the introduction of calorie labels?”* (rated on a five-point scale from *Very unhappy* to *Very happy* with an additional option of choosing *Didn't notice the labels*); and *“Would you like calorie labels to remain in place permanently?”* (rated on a five-point scale from *No, definitely not* to *Yes, definitely*); and
(4) *Compliance with the study protocol.* Compliance visits were conducted at each of the three sites on the first day of intervention when non-compliant items, *i.e.* unlabelled products, were noted. Thereafter, fortnightly compliance visits were carried out at each site; protocol violations were recorded and rectified in discussion with the cafeterias' management teams.

#### 2.4.2 Intervention impact

##### 2.4.2.1 Primary outcome

Total energy (kcal) purchased daily from intervention items, controlling for the total transactions as measured from daily sales records.

##### 2.4.2.2 Secondary outcome

Number of items purchased daily from (a) intervention items, and (b) non-intervention items, controlling for the total transactions.

##### 2.4.2.3 Other measures

Covariates recorded in the study and considered in analyses: total number of transactions per day (to control for daily footfall in each site); day of week; and weather conditions (daily average temperature).

### 2.5 Data analysis

#### 2.5.1 Feasibility and acceptability

Feasibility and acceptability indicators were summarised using descriptive statistics. Qualitative assessments gathered via semi-structured interviews with worksite managers and caterers were coded and summarised narratively.

#### 2.5.2 Intervention impact

Analyses were conducted in R.3.4.2. Our protocol and trial registration pre-specified that we would use generalised linear mixed models (GLMM) to examine the impact on total energy (kcal) purchased per day from intervention items controlling for the total transactions, adjusted for time trends (using day relative to the intervention start date as a random slope per site) and with random effects for worksite. However, an examination of the data showed considerable heterogeneity in variances between the three sites. Various variance-stabilising transformations - including logarithmic and square-root transformations - were investigated but none proved adequate. Therefore, due to heteroscedasticity, both the mean and variance of parameters were included (using identity and log links respectively) in the more general analysis framework of a Generalized Additive Model for Location, Scale and Shape (GAMLSS) mixed model (Rigby & Stasinopoulos, 2005; Stasinopoulos & Rigby, 2007). This allowed explicit parameters for site-variances to take different values.

Uncharacteristic days, such as days showing large changes in energy purchased due to special events at the worksites, were included as dummy variables to allow for an unbiased estimate of the intervention effect (more details on this can be found in the Results section). Site was fitted as a random effect as per protocol. We also fitted parameters when necessary for separate variances: (i) on different weekdays; and (ii) different sites. The model diagnostics ranged from acceptable to good. Additional sensitivity analyses were conducted to explore whether partial compliance with the intervention affected the obtained results.

## 3 Results

### 3.1 Feasibility and acceptability

Of the four worksites approached, all were eligible to participate. Three sites were recruited and received the labelling intervention. All three recruited worksite cafeterias successfully completed the baseline and intervention periods (attrition rate of 0%), attesting to the feasibility of retaining eligible worksites (see also Fig. 1 CONSORT diagram).

Implementation of the intervention proved feasible, with the proportion of items that were labelled being above 80%: 83% at Site 1, 94% at Site 2, and 85% at Site 3.

Cafeteria patrons who took part in the post-study survey strongly supported the intervention. The survey was completed by 250 employees, approximately 8.5% of the total number of employees based at the three worksites. A large proportion of respondents (83%) were either happy or very happy about the introduction of calorie labelling, 12% were neither happy nor unhappy, 1% were unhappy or very unhappy, whilst 2% reported not noticing any changes in labelling. Furthermore, the vast majority of surveyed employees (87%) reported that they would like calorie labelling to remain in place permanently, answering either *Yes, definitely* or *Yes, probably*, 10% didn't mind, whilst only 1% objected to calorie labelling remaining in place permanently, answering either *No, probably not* or *No, definitely not*.

The Box in the Online Supplementary Materials summarises the themes identified in the thematic analysis of the post-intervention interviews conducted with worksite managers. As in the previous study (Vasiljevic et al., 2018), worksite managers were receptive and supportive of the intervention, seeing the calorie labels as a positive addition to the cafeteria, rather than taking something away from patrons. In the current study, managers again commented that the initial implementation of calorie labelling was labour-intensive and time-consuming, but once this was done the intervention was simple to maintain. Managers reported positive feedback from their patrons and, in contrast to our previous study, the managers also noted that patrons commented on the clarity of the visual display of the energy content on the labels used for this study, demonstrating that at least for the employees who took part in the post-study survey, the labelling intervention tested in this study was more prominent and more noteworthy when compared to the calorie labelling intervention used in the prior study. Managers also reported that patrons expressed mixed feelings towards the presentation of calorie information. Some patrons thought this made their food choices easier, whereas others felt that additional nutritional information may be needed to help them make more informed dietary choices (seeMozaffarian, 2017). Furthermore, managers also highlighted the benefits of setting up calorie labelling in their cafeterias with the view of aiding their employees’ dietary choices. Finally, managers hoped that the independent evaluation of the calorie labelling intervention would help them to set-up calorie labelling initiatives which may, at some point in the future, be mandated through government policy (Department of Health and Social Care, 2018).

Compliance with the study protocol varied across sites and products. A detailed record of items that were non-compliant at each site and the dates when these were then labelled as per protocol can be seen in Table S1 in Online Supplementary Materials. Sensitivity analyses were performed to check for differences in the effects of the intervention between days when all items were compliant and when they were not.

### 3.2 Intervention impact

**Fig. 4:**
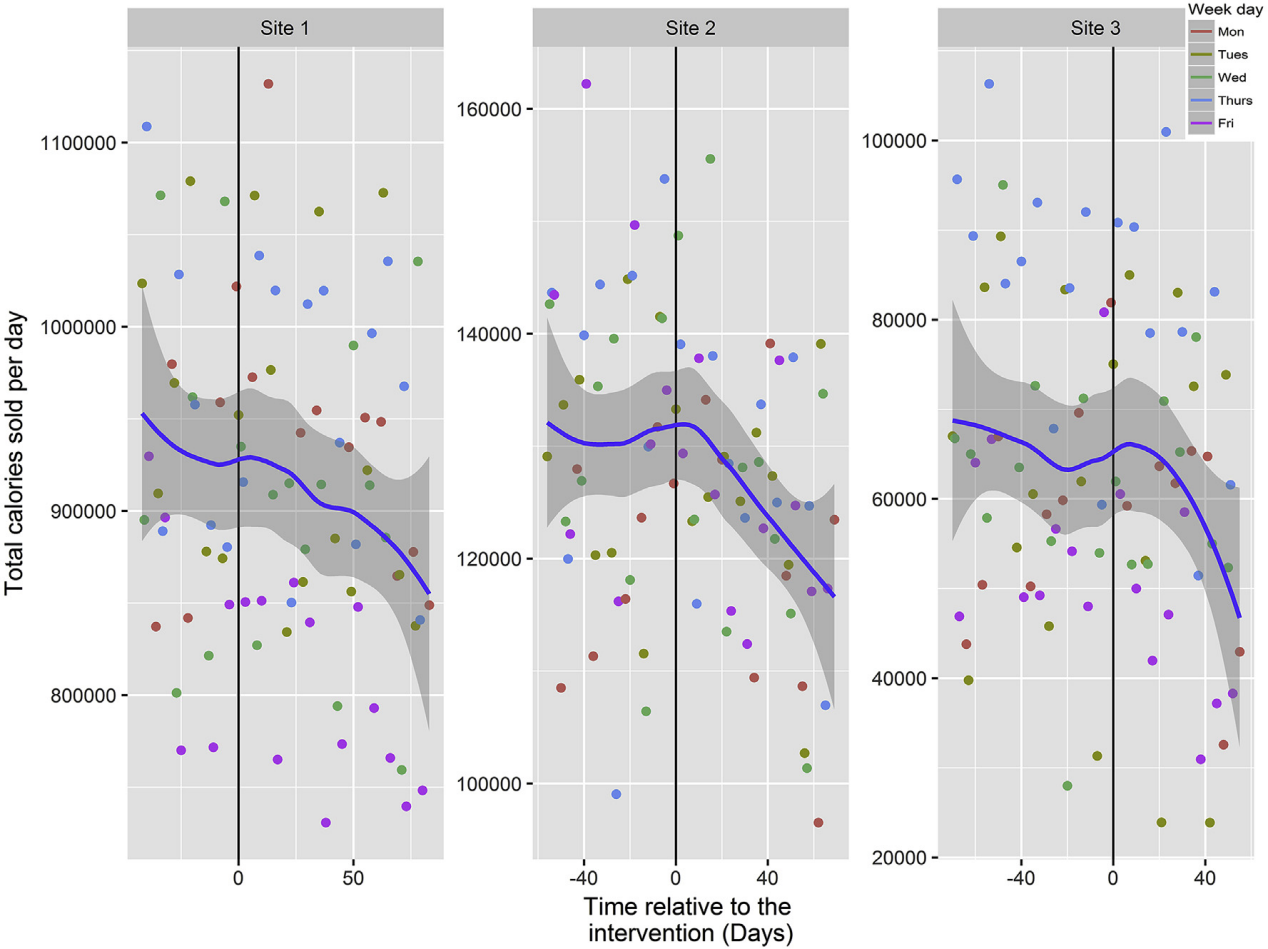
Total energy sold per day for intervention items across the three sites with information displayed for day of the week.

**Fig. 5:**
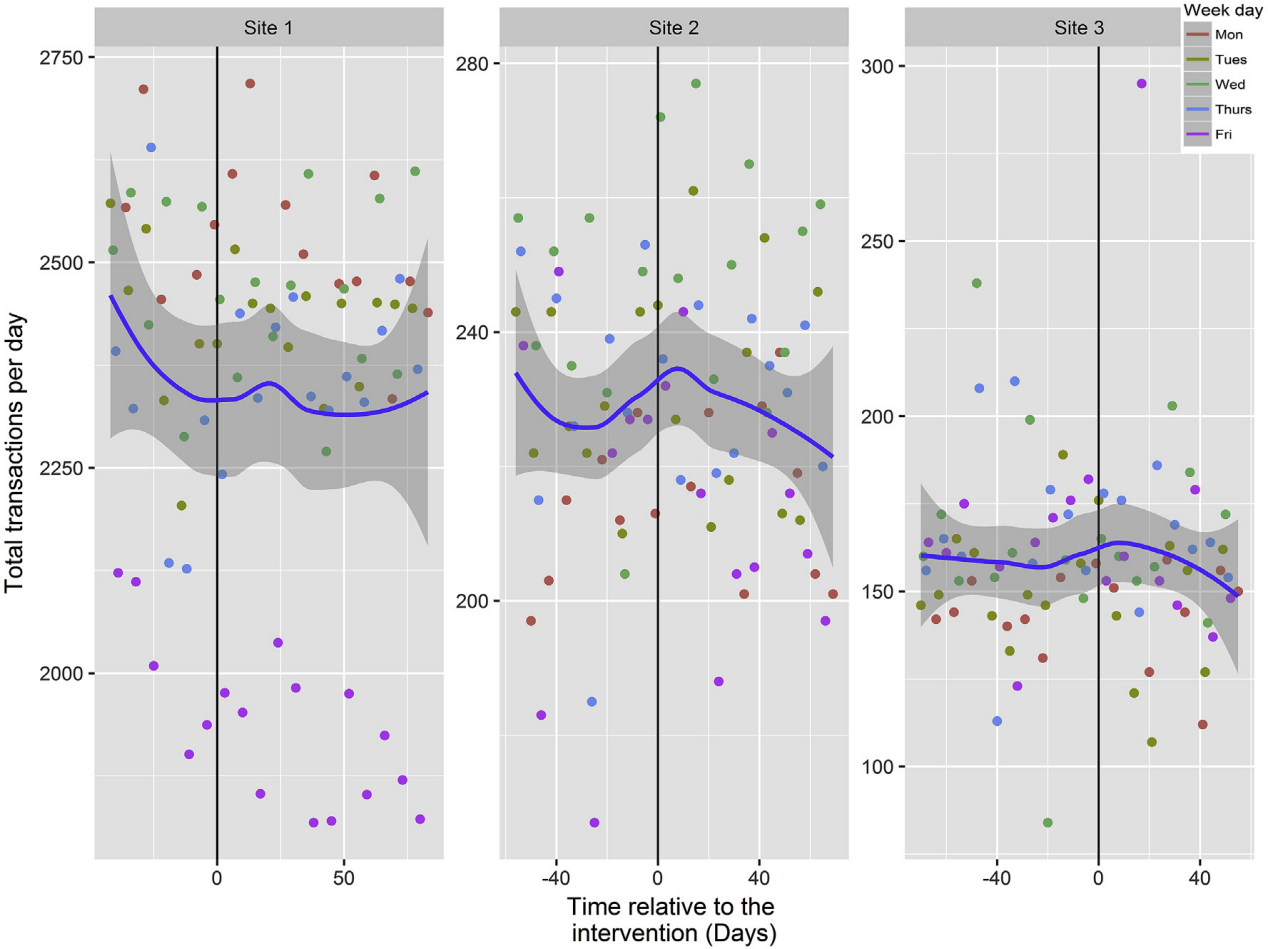
Transactions per day for intervention items across the three sites with information displayed for day of the week.

An examination of the plots for total energy purchased from intervention items and the number of transactions at each site showed different underlying trends at different sites (see Fig. 4, Fig. 5). The graphical presentation of the data in Fig. 4, Fig. 5 uses best fit lines based on loess curves, making minimal assumptions about the data. As can be seen in Fig. 4, Fig. 5 there were: (i) strong weekday effects with, for example, at all sites more energy being purchased on Thursdays, and at Site 1 less energy purchased across fewer transactions on Fridays; and (ii) special features in some of the sites that had to be accounted for by dummy variables. For example, at Site 3 there were three days on which a free buffet was available in the cafeteria, one day with a free BBQ on offer, and one day with a non-free BBQ for which employees had to purchase a ticket. A dummy variable indicating these five special events was included as a control variable in the statistical modelling of the primary outcome.

Given the small number of sites, there was limited scope to include explanatory terms in the modelling. The final model included the following covariates: number of transactions, time relative to the intervention, week-day, daily average temperature, and a dummy variable denoting the five special events at Site 3. Model diagnostics - *i.e.,* residual plots, autocorrelation – ranged from acceptable to good. Alternative models were also examined (see sensitivity analysis below).

### 3.3 Primary outcome

Pooling the data across the three sites showed no significant effect of prominent calorie labelling on daily energy purchased: −0.6% [95%CI -2.5 to 1.2, *p* = .487, *M* = −2410.2 (*SD* = 5992.6) total daily calories]. By-site analyses showed similar null effects at each of the three sites: Site 1 (−0.4% [95%CI -1.2% to 0.4%, *p* = .299, *M* = −3896.2 (*SD* = 6482.3) total daily calories]); Site 2 (0.3% [95%CI -4.5% to 5.1%, *p* = .890, *M* = 444.3 (*SD* = 5543.2) total daily calories]); and Site 3 (−7.4% [95%CI -16.5% to 1.7%, *p* = .110, *M* = −4891.8 (*SD* = 5287.4) total daily calories]). The model estimates are shown in Table 3. A sensitivity check where we excluded the dummy variable for special events replicated these results.

**Table 3:** Primary analysis of total daily energy purchased.

	Calories	95%CI	p	Pre-Intervention Mean	% Change	95% CI
	M (SD)			Daily Calories	Post-intervention	
Overall model						
Modelling of the mean (identity link):						
(Intercept)	43742.9 (8625.1)	(33982.9, 53502.9)	<0.0001			
Day relative to intervention	125.0 (107.9)	(2.9, 247.1)	0.0458			
Transactions	376.7 (7.0)	(368.8, 384.5)	<0.0001			
Week day (Ref = Monday)		(-2213.9, 8462.6)				
Tuesday	3124.4 (4717.5)		0.2525			
Wednesday	−4084.109 (4443.1)	(-9111.9, 943.7)	0.1127			
Thursday	8091.4 (4652.6)	(2826.5, 13356.2)	0.0029			
Friday	4721.3 (5865.9)	(-1916.5, 11359.1)	0.1646			
Temperature	−1524.5 (585.5)	(-2187.0, −862.0)	<0.0001			
Special Event	−18313.4 (12497.0)	(-32454.8, −4171.9)	0.0118			
Intervention	−2410.2 (5992.6)	(-9191.3, 4371.0)	0.4867	374551.9	−0.6%	(-2.5%, 1.2%)

Modelling of the variance (log link):						
(Intercept)	11.155 (0.198)	(10.931, 11.379)	<0.0001			
Week day (Ref = Monday)						
Tuesday	0.092 (0.239)	(-0.179, 0.363)	0.5052			
Wednesday	−0.015 (0.181)	(-0.220, 0.189)	0.8842			
Thursday	0.017 (0.225)	(-0.237, 0.272)	0.8934			
Friday	0.359 (0.259)	(0.066, 0.652)	0.0172			
Site (Ref = Site 1)						
Site 2	−2.114 (0.201)	(-2.342, −1.886)	<0.0001			
Site 3	−1.532 (0.213)	(-1.772, −1.291)	<0.0001			

By-site						
Modelling of the mean (identity link):						
(Intercept)	39965.7 (4569.9)	(29612.6, 50318.9)	<0.0001			
Day relative to intervention	73.0 (64.5)	(-44.6, 190.6)	0.224766			
Transactions	376.9 (8.0)	(363.1, 390.7)	<0.0001			
Week day (Ref = Monday)						
Tuesday	2320.3 (1498.1)	(-3148.7, 7789.4)	0.406505			
Wednesday	−4615.0 (3340.7)	(-9762.9, 532.9)	0.080204			
Thursday	7701.1 (10391.2)	(2267.7, 13134.5)	0.005912			
Friday	4342.4 (2886.6)	(-2439.8, 11124.6)	0.210756			
Temperature	−1287.7 (364.1)	(-1975.8, −599.6)	0.000303			
Special Event	−13141.4 (717572.0)	(-28184.6, 1901.9)	0.088184			
Intervention:						
Site 1	−3896.2 (6482.3)	(-38047.7, 30255.3)	0.29893	927358.1	−0.4%	(-1.2%, 0.4%)
Site 2	444.3 (5543.2)	(-6244.5, 7133.1)	0.88971	130320.0	0.3%	(-4.5%, 5.1%)
Site 3	−4891.8 (5287.4)	(-13340.6, 3557.1)	0.11040	65977.7	−7.4%	(-16.5%, 1.7%)

Modelling of the variance (log link):						
(Intercept)	11.264 (0.293)	(10.959, 11.569)	<0.0001			
Week day (Ref = Monday)						
Tuesday	0.131 (0.269)	(-0.144, 0.407)	0.351			
Wednesday	−0.016 (0.253)	(-0.327, 0.294)	0.918			
Thursday	0.037 (0.314)	(-0.240, 0.314)	0.794			
Friday	0.339 (0.296)	(0.029, 0.649)	0.033			
Intervention:						
Site 1	−0.194 (0.285)	(-0.515, 0.127)	0.238			
Site 2	−0.046 (0.278)	(-0.411, 0.318)	0.803			
Site 3	0.057 (0.284)	(-0.290, 0.404)	0.748			
Site (Ref = Site 1)						
Site 2	−2.202 (0.354)	(-2.586, −1.818)	<0.0001			
Site 3	−1.674 (0.321)	(-2.035, −1.312)	<0.0001			

*Note.* 95%CI based on the likelihood ratio test.

### 3.4 Sensitivity analysis

A sensitivity analysis was conducted in which all items non-compliant with the labelling intervention at any point during the intervention phase were excluded from the calculation of the total calories per day. This led to the removal of 44 (9.8%), 5 (1.7%) and 30 (10.1%) products at Sites 1, 2 and 3, respectively. Similar results were obtained to those using the primary models: there was no overall effect of the intervention −1.2% [95%CI -3.2% to 0.8%, *p* = .240, *M* = −4079.4 (*SD* = 5992.1) total daily calories]. Unlike in the primary analysis, the impact of the calorie labelling intervention on energy purchased was statisticially significant at Site 3 when compliance was accounted for: −29.0% [95%CI -47.7% to −10.2%, *p* = .003, *M* = −12958.8 (*SD* = 7410.9) total daily calories]. These estimates should be considered with caution due to the particularly large confidence intervals obtained for energy purchased at Site 3. The impact of the prominent calorie labelling was not statistically significant in the other two sites when taking into consideration the non-compliant items: Site 1 (−1.5% [95%CI -5.8% to 2.7%, *p* = .481, *M* = −12933.2 (*SD* = 31705.3) total daily calories]; Site 2 (−0.5% [95%CI -6.1% to 5.0%, *p* = .851, *M* = −685.6 (*SD* = 6315.5) total daily calories]).

### 3.5 Secondary outcome

Our secondary outcome consisted of modelling the total number of (a) intervention items, and (b) non-intervention items sold per day since it was not possible to model the total daily energy for non-intervention items separately. Daily number of transactions, day of the week, and daily average temperature served as covariates as in the primary outcome models.

#### 3.5.1 Intervention items only

There was no overall effect of labelling on total sales of intervention items per day [15.2 items (*SD* = 35.7) (95%CI -25.2 to 55.6), *p* = .460]. There was also no impact on total sales of intervention items per day in the individual sites.

#### 3.5.2 Non-intervention items only

There was no overall effect of the intervention on total sales of non-intervention items per day [0.5 items (*SD* = 5.0) (95%CI -5.2 to 6.1), *p* = .867]. The by-site analysis showed a statistically significant decrease in daily sales of non-intervention items following the introduction of calorie labelling at Site 1 [-44.8 items (*SD* = 29.3) (95%CI -77.9 to −11.7), *p* = .009]*.* The other two sites did not demonstrate a statistically significant effect of the intervention on daily sales of non-intervention items: Site 2 [2.8 items (*SD* = 5.2) (95%CI -3.1 to 8.7), *p* = .358]; Site 3 [-4.5 items (*SD* = 5.7) (95%CI -10.9 to 2.0), *p* = .174]).

## 4 Discussion

Recruitment and retention of worksite cafeterias in the present pilot trial proved feasible. Post-intervention feedback suggested high levels of intervention acceptability amongst both patrons and catering staff, with 87% of cafeteria patrons wanting the prominent calorie labelling to remain in place. In terms of intervention effectiveness, pooling the data across the three sites showed no effect of the intervention on daily energy purchased: −0.6% [-2.5%, 1.2%]. Modelling the impact of the intervention at each individual site showed similar null effects.

The overall non-significant effect found across sites (−0.6%) replicates the overall size of effect of calorie labelling obtained in our prior pilot trial (−0.4%) (Vasiljevic et al., 2018). Together, these results suggest that the synthesised effect size estimates of the potential impact of calorie labelling in recent systematic reviews (Crockett et al., 2018; Zlatevska et al., 2018) may be an overestimate of the true effect found in general populations in real world settings. The estimated effect size of −7.8% from calorie labelling on menus presented in the recent Cochrane Review was based on three US-based experimental studies, two of which were conducted in the same university cafeteria (Crockett et al., 2018). This evidence was rated of low quality using GRADE assessment criteria, meaning that the estimated effect size is likely to change with more evidence (Crockett et al., 2018). The estimate of −4.6% provided in the larger systematic review by Zlatevska and colleagues (Zlatevska et al., 2018) was based mainly on studies conducted in the USA, often carried out in university establishments and testing the effects of calorie labelling amongst university staff and students, often under controlled laboratory settings. The effect of calorie labelling in Zlatevska's review was shown to be larger in laboratory settings than in field studies (Zlatevska et al., 2018). Furthermore, a narrative synthesis of evidence suggests that calorie labelling may generate larger effects amongst those in higher socio-economic positions (SEPs) (Sarink et al., 2016), the populations on which much of the evidence in the Cochrane Review and Zlatevska's review is based.

Post-hoc power analyses suggest that our present study was powered to detect an effect size of 5.23% (two-tailed). We were therefore powered to detect an effect of the size suggested by the recent Cochrane systematic review (Crockett et al., 2018), which is arguably the closest estimate of effect size relevant for the current study given the synthesised effect was based solely on randomised experimental evidence in field settings. An as yet unexplored moderator of these effects is the country in which studies were conducted. Our two field cafeteria experiments – conducted in England – have thus far yielded smaller and statistically non-significant effects in contrast to field cafeteria experiments conducted in the USA.

Within-site analyses in both the present and our previous studies (Vasiljevic et al., 2018) suggest that calorie labelling has heterogeneous effects in different worksite establishments which may reflect differences in participants’ characteristics. However, due to the small number of sites in both the previous and current studies (*n* = 9), we were not able to formally examine demographic characteristics of participants at each site as a potential moderator of the effects of calorie labelling.

Decisions about the introduction of calorie labelling may rest upon considerations other than evidence of effectiveness to reduce energy purchased or consumed. The high levels of acceptability of the prominent calorie labelling and high levels of support for its continuation amongst worksite managers, catering staff, and cafeteria patrons are in line with evidence showing that the public consider information provision or education as acceptable interventions to change dietary behaviour (Diepeveen, Ling, Suhrcke, Roland, & Marteau, 2013). This is consistent with growing demands from consumers for information about their food, whether about nutritional content, allergens, or provenance (Roberto et al., 2009). A further indirect effect of calorie labelling – not assessed in the current study – is its potential impact on reformulation of products or the range of menu options provided. An additional analysis by Zlatevska and colleagues of 41 studies that measured the impact of mandatory calorie labelling on retailers’ food offering, estimated that after the introduction of calorie labelling, retailers offered 15 calories less per meal (Zlatevska et al., 2018). In the context of randomised controlled trials such as those reported here, these effects are excluded by careful manipulation only of the labelling itself and not the product range. Thus, even though the direct impact of calorie labelling on consumer purchasing may be smaller than previously estimated, there may be additional indirect effects if implemented in routine practice, which may result in a reduction in the energy content of foods offered for purchase and consumption. Reformulation of products or changes in menu options may also lead to improvements in the nutritional quality of the foods available, through reductions in saturated fat, free sugars or sodium and/or increases in fruit and vegetable content, bringing additional beneficial health impacts (see Mozaffarian, 2017; Shangguan et al., 2019; Swinburn et al., 2019).

### 4.1 Strengths and limitations

One notable strength of the present study is the use of prominent calorie labels designed to maximise readability following a scoping literature review. Furthermore, in the present study we worked closely with the three worksite catering teams in order to improve their data-capture methods prior to study commencement. We also carried out fortnightly fidelity checks at all sites, which enabled us to rectify any issues with intervention implementation and data capture in a timelier fashion than was possible in our previous study (Vasiljevic et al., 2018). These changes to the protocol and intervention design resulted in higher quality data, lending greater confidence in any conclusions that could be drawn from the present study.

The above strengths notwithstanding, the study was limited in several respects. The most notable limitation was the small number of participating sites and their heterogeneity. Since this was a pilot trial, we tested the prominent calorie labels and improved protocol amongst three sites, which was the maximum number of sites that we could realistically recruit and set-up the intervention in the given time period. Another limitation of this pilot study was that we were only able to recruit the required three sites by approaching four worksites, which were members of a Healthy Eating in the Workplace Advisory Group. The feasibility of recruiting a larger number of potentially more diverse worksite cafeterias for a larger trial is unknown. However our other feasibility measures show that when workplaces are willing to try this intervention it is possible to deliver the intervention successfully and collect the data necessary for evaluation. The study was further limited by using energy purchased as a proxy for consumption. Purchasing does not take into account possible food waste, food bought and consumed from other establishments, and food freely available at the worksites. However, this is likely to apply equally to both intervention and control periods and should therefore not impact the estimates of energy purchased across different study periods. Future studies could improve estimates of food consumption by measuring food waste and establishing a protocol to measure and control for consumption of food obtained from outside the worksite cafeteria setting.

### 4.2 Future research directions

Although recent systematic reviews suggest that calorie labelling has an impact on energy selected or purchased (Crockett et al., 2018; Shangguan et al., 2019; Zlatevska et al., 2018), they each highlight the paucity of well-controlled experimental studies in field settings, with one review suggesting that the effect of calorie labelling is weaker in field compared with laboratory settings (Zlatevska et al., 2018). Future research should therefore aim to estimate the impact on selection and consumption of calorie labelling in field settings in robust studies using experimental designs. Aside from the current study and our prior study (Vasiljevic et al., 2018), all other existing experimental field studies have been conducted in the US. More studies outside of the US are therefore needed to examine the generalisability of calorie labelling effects beyond the US.

Even though recent reviews (Shangguan et al., 2019; Zlatevska et al., 2018) have found no significant difference between simple calorie labels *vs.* enhanced labels – such as physical activity calorie equivalents [PACE] labels or pictorial warning labels - these supplementary analyses were based on limited evidence generated in laboratory settings. Further research is warranted to test such enhanced calorie labelling using robust experimental designs in field settings to estimate the potential for such labels to reduce the energy of food selected or consumed.

Diet-related disease is linked both to overconsumption of energy and to the nutrient composition of the diet. The recent systematic review by Shanguann and colleagues (Shangguan et al., 2019) found no significant difference in the impact on consumption of calorie labelling *vs.* nutritional labelling of specific nutrients. However, the moderation analyses were based on a limited number of studies, suggesting that the estimate of this effect may change when there is a larger evidence base to probe this difference. Future studies could also consider whether additional labelling of specific nutrients has greater impact on food consumption than calorie labelling alone.

### 4.3 Policy implications

While studies to date do not provide a reliable population level estimate of the potential for calorie labelling to reduce energy purchased out-of-home, any decision to introduce, or even mandate, calorie labelling should take into consideration a range of other factors. First, such information is valued by consumers (Roberto et al., 2009). Second, there is some evidence that mandatory calorie labelling could have positive supply-side effects through product and menu reformulation (Zlatevska et al., 2018). Given that increasing the availability of lower energy foods in worksite cafeterias can reduce energy purchased (Pechey et al., 2018) this could be an effective route through which calorie labelling could contribute to tackling obesity.

## 5 Conclusions

There was no evidence that prominent calorie labelling changed daily energy purchased across three English-based worksite cafeterias. The intervention was feasible to implement and acceptable to patrons and managers.

## Ethics approval

Approved by the Psychology Research Ethics Committee of the University of Cambridge (Reference Number: PRE.2016.035).

## Availability of data and materials

The data are commercially sensitive, provided by the participating worksites on condition that they are not shared beyond the research team.

## Conflicts of interest

The authors declare that they have no competing interests.

## Funding

The study was funded by the National Institute for Health Research Policy Research Programme (Policy Research Unit in Behaviour and Health [PR-UN-0409-10109]) and the Institute of Grocery Distribution [RG83425]. RP is supported by a Wellcome Trust Research Fellowship in Society and Ethics [106679/Z/14/Z]. SAJ is supported by the Oxford NIHR Biomedical Research Centre and the Oxford NIHR Collaboration for Leadership in Applied Health Research and Care (CLAHRC). The funders had no role in the study design, data collection, analysis, or interpretation. The views expressed in this publication are those of the authors and not necessarily those of the funders, the NHS, the National Institute for Health Research, the Department of Health and Social Care or its arm's length bodies, and other Government Departments.

## Authors’ contributions

All authors collaborated in designing the study. MV and GF coordinated the intervention implementation and data collection. MP performed the data analyses. MV drafted the manuscript, GF, MP, GJH, RP, SAJ, and TMM provided critical revisions to the manuscript. All authors read and approved the final manuscript.
