# Supplementary material for: What is the impact of increasing the prominence of calorie labelling? A stepped wedge randomised controlled pilot trial in worksite cafeterias
Source: Appetite. 2019 Oct 1;141:104304. doi: 10.1016/j.appet.2019.05.035 (PMC8161726; doi:10.1016/j.appet.2019.05.035)
Supplement: Multimedia component 1 [file mmc1.docx]

**Online Supplementary Materials**

**What is the impact of increasing the prominence of calorie labelling? A stepped wedge randomised controlled pilot trial in worksite cafeterias**

Milica Vasiljevic^1,2^, Georgia Fuller^1^, Mark Pilling^1^, Gareth J. Hollands^1^, Rachel Pechey^1^, Susan A. Jebb^1,3^, & Theresa M. Marteau^1^

^1^Behaviour and Health Research Unit, Institute of Public Health, University of Cambridge, Cambridge, UK

^2^Department of Psychology, Durham University, Durham, UK

^3^Nuffield Department of Primary Care Health Sciences, University of Oxford, Oxford, UK

Calorie Labelling: How to design your labels pp.2-9

Box: Themes identified from semi-structured interviews with worksite managers pp.10-11

Table S1: List of non-compliant items pp.12-14

**Calorie Labelling: How to design your labels**

Background

Thank you for participating in IGD’s Healthy Eating programme. This document is prepared by the University of Cambridge (UoC) and provides instructions for displaying calorie labels in your cafeteria. Please use this to prepare your labels containing calorie information for all products at point of choice.

**You will introduce calorie labels from [DATE]**

Ahead of implementation, we will be in touch to arrange a convenient time to view these labels before they are finalised. In the meantime, please get in touch if you have any questions.

Contact details

Georgia Fuller
Research Assistant
Behaviour and Health Research Unit
University of Cambridge

Email: [email here]
Telephone: [telephone here]

Where to put labels

Calorie information should be directly above, below or beside the product. Where this is not possible please inform UoC to discuss and agree a solution.

- Labels on products – see Figures 1A and 1B
- Shelf-edging at point of choice – see Figure 2
- Tent cards next to products – see Figure 3
- Menus (printed or electronic via email or screens) – see Figures 4 and 5

Which products to label

All products within the cafeteria should be labelled with calorie information, including:

- Main meals (including side dishes)
- Snacks (including all confectionery, crisps, sandwiches, protein pots etc.)
- Breakfast selection (both hot and cold options)
- Cold drinks
- Condiments (portioned)

Salad bars, hot drinks and vending are **excluded** from the study and do not need additional labelling. However, please note you will still need to send daily sales information for salad bars to UoC.

*Other areas where calorie labelling may be difficult (such as deli bars) will be dealt with case-by-case by UoC.*

Label Content

*The label should include:*

- Name of food or drink item
- Calorie content written as ‘XXX CALORIES’
- Portion size if relevant (e.g. per slice, per ladle, per average bowl/serving if pre-portioned or served to the customer)
- Price

*The label should* ***not*** *include:*

- Any additional information such as Reference Intakes, which should be removed
- Any alternative terms to ‘calories’ – e.g. do not use ‘kcal’ or ‘kJ’

**If applicable, allergen information should continue to be provided as usual.**

Label Design

Labels should be legible and prominent to the customer (from where they will be standing at point of choice). To ensure this, the calorie content, e.g. ‘**120 CALORIES**’, should be:

- Bolded
- Written in uppercase
- Written in Verdana typeface
- Written in:
  1. minimum font size 14 for product labels, shelf-edging and tent cards
  2. minimum font size 18 for A4 daily menus (please note, weekly menus will be designed on a case-by-case basis)
- Written two font sizes larger than the rest of the font on the label (calorie information should appear larger than the product name, price and portion size)
- Written in black typeface on a white background (if coloured backgrounds are used, please contact us to discuss options)
- Written horizontally on the label with as much white space around the text as possible

Please see the Appendix to find examples (Figures 1A, 1B, 2, 3, 4 and 5).

**Appendix**

**Figure 1A -** *Example of a calorie label on a product:*


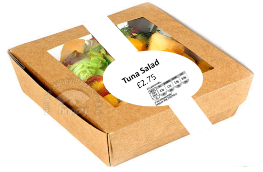


This is not correct as full Reference
Intake (RI) information is displayed
instead of Calories.


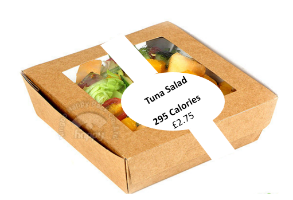


This is correct.


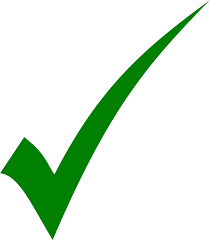


Tuna Salad
 **295 CALORIES** £2.75

**Figure 1B -** *Example of a calorie label on a product:*

*N.B. to demonstrate what labels may look like if allergen information would usually be present, this has been included in one of the example product labels below. For the purposes of this study, if allergen information is not already on your labels, please do not include it.*

***Option 1.*** *Calorie label without allergen advice*

Cherry Muffin

**651 CALORIES**

90p

***Option 2.*** *Calorie label with allergen advice*

Cherry Muffin

**651 CALORIES**90p

ALLERGY ADVICE

**WHEAT, EGGS, MILK**

**Figure 2 -** *Example of a calorie label on shelf-edging:*

Cherry Muffin

**651 CALORIES**90p

**Figure 3 -** *Example of a calorie label on a tent card:*

Cherry Muffin

**651 CALORIES**

90p

**Figure 4 -** *Example of calorie labelling on a daily menu:*

Monday’s Menu

| Cumberland Sausages | **150 CALORIES** | £2.95 |
| --- | --- | --- |
| Cajun Chicken | **208 CALORIES** | £2.95 |
| Cheese & Onion Pasties | **433 CALORIES** | £1.60 |
| Leek & Potato Soup | **193 CALORIES** | £0.80 |
| Sweetcorn | **95 CALORIES** | £0.50 |
| Side Salad | **11 CALORIES** | £0.50 |

**Figure 5 -** *Example of calorie labelling on a weekly menu:*

|  | **Monday** | **Tuesday** | **Wednesday** | **Thursday** | **Friday** |
| --- | --- | --- | --- | --- | --- |
| **House Soup**      **‘Real’ Soup** | Carrot & Coriander (v) **104 CALORIES**    Seafood Broth  **240 CALORIES** | Tomato and Basil (v)  **112 CALORIES**  Chicken and Mushroom  **129 CALORIES** | Leek & Potato (v)  **137 CALORIES**    Spicy Chickpea (v)  **217 CALORIES** | Tomato (v)  **98 CALORIES**    Cream of Vegetable (v)  **217 CALORIES** | Cauliflower (v)  **108 CALORIES**    Minestrone (v)  **194 CALORIES** |
| **Our ‘made to order’ Pizza Menu is available every day** | | | | | |
| **Al Forno** | Pork Chop  **249 CALORIES** | Spicy Bean Burger with Cheese (v)  **580 CALORIES** | Goats Cheese & Red Onion Tart (v)  **275 CALORIES** | Chicken & Egg Fried Rice Pot  **390 CALORIES** | Beef Chilli with Wedges  **410 CALORIES** |
| **Nutritional Selection** | Chickpea Curry & Naan (v)  **240 CALORIES** | Kedgeree  **404 CALORIES** | Sardines with  Tomato Stew  **244 CALORIES** | Vegetable Pasta Bake (v)  **290 CALORIES** | Feta, Roast Vegetable & Quinoa Pot (v)  **191 CALORIES** |
| **Main Course** | Chicken Supreme & Gravy  **228 CALORIES** | Cumberland Pie with Mash  **400 CALORIES** | Beef & Guinness Pie  **332 CALORIES** | Roast Lamb & Gravy  **294 CALORIES** | Battered Haddock Fillet  **326 CALORIES** |
| **Sides**  **Starch**  **Veg** | Chips  **260 CALORIES** | Chips  **260 CALORIES** | Patatas Bravas  **184 CALORIES** | Roast Potatoes  **149 CALORIES** | Chips  **260 CALORIES** |
|  | Steamed Carrots  **41 CALORIES** | Cauliflower Cheese  **138 CALORIES** | Peas & Corn  **65 CALORIES** | Parsnips  **179 CALORIES** | Peas  **81 CALORIES** |
|  | Corn on the Cob  **155 CALORIES** | Green Beans  **31 CALORIES** | Steamed Broccoli  **31 CALORIES** | Cabbage  **27 CALORIES** | Vegetable Medley  **50 CALORIES** |
| **Hot Dessert** | Coffee Sponge  **230 CALORIES**  Custard  **190 CALORIES** | Rice Pudding  **111 CALORIES**  Jam Sauce  **75 CALORIES** | Bread Pudding  **360 CALORIES**  Custard  **122 CALORIES** | Orange Cake  **198 CALORIES**  Custard  **122 CALORIES** | Ginger Sponge  **355 CALORIES**  Toffee Sauce  **132 CALORIES** |

**Box: Themes identified from semi-structured interviews with worksite managers.**

| Themes | Subthemes | Comments |  |
| --- | --- | --- | --- |
| Information provided | Clear Information† (2 of 3 sites) | *“It was very clear, big, bold writing”* |  |
|  |  |  |  |
|  |  | *“Just the calories, rather than doing the whole thing, the fat, the protein, the carbs. It just gives a very clear message”* |  |
|  |  |  |  |
|  |  | *“The previous ones we put calories and kilojoules …I think everybody is more aware of calories so it was a clearer message”* |  |
|  |  |  |  |
|  | Missing Information† (2 of 3 sites) | *“Some people question about the fact whether we should just be saying about calories, whether we should be giving more dietary information around ingredients”* |  |
|  |  |  |  |
|  |  | *“There's been a few suggestions around whether it is productive towards a...balanced diet, talking about just calories”* |  |
|  |  |  |  |
|  |  | *“…and then there's the sugar as well because some of the things that have low calorie have higher sugar”* |  |
|  |  |  |  |
|  | Eye-catching Design (2 of 3 sites) | *”I think it is probably more effective than previous ones I've been involved in, in terms of it being a little more bolder than the previous times which I think is useful”* |  |
|  |  |  |  |
|  |  | *“If it was much smaller people probably wouldn’t pay attention (to) what’s on the label where it was quite big and bold so you can see, it is one of the first things that you can see when you look at the label so that was good”*  *“I understand that part of the study was for (the labelling) to be so big; I think from a customer point of view it looked a bit ridiculous if I’m honest”* |  |
|  |  |  |  |
|  | Awareness of Information (2 of 3 sites) | *“People were talking about it so they definitely noticed the difference”* |  |
|  |  |  |  |
|  |  |  |  |
| Implementation | Time-consuming† (3 of 3 sites) | *“It was more work at the beginning getting all the labelling done”* |  |
|  |  |  |  |
|  |  | *“It was hard work obviously but…it’s not (a) major change to my everyday tasks”* |  |
|  |  |  |  |
|  |  | *“There was quite a lot of work for (the catering team) first off but once that was done then…it was all there.”* |  |
|  |  |  |  |
|  | Overcoming challenges (3 of 3 sites) | *“We had to get extra information from the supplier and if the supplier didn’t have it we had to go...to the actual manufacturer”* |  |
|  |  |  |  |
|  |  |  |  |
|  |  | *“Some things were challenging for us in terms of getting some of the information from suppliers”* |  |
|  |  |  |  |
|  | Feeling Supported (3 of 3 sites) | *“(The University of Cambridge has) been very helpful with it, you’ve smoothed over where there's times where the managers would have been a bit more stressed if they didn’t have your support”* |  |
|  |  |  |  |
|  |  | *“You guys (did) a lot of work actually, you helped me a lot and for me it was just making sure that everything was in place really.”* |  |
|  |  |  |  |
|  |  | *“What I felt was really good this time was that there was a continual visitation to site from (University of Cambridge). That, I think, helped keep the impetus and also helped in terms of potentially going off-piste"* |  |
|  |  |  |  |
|  | Rationale (2 of 3 sites) | *“We wanted to do it so that we can learn from the experience of doing it so if it does become [law] later on...we've trialled it in our own area and got a better understanding of it”* |  |
|  |  |  |  |
|  |  | *“It’s something that’s helped be a foundation of our health and well-being ambitions and drives within the business so it’s been good”* |  |
|  |  |  |  |
|  |  | *“...it surprises me that more people aren’t interested in the nutritional value of what they're putting into their bodies. I think it’s great that we’ve offered that information..."* |  |
|  |  |  |  |
|  | Reactions to Calorie Content (2 of 3 sites) | *“Most people were quite surprised to see the amount in calories in certain foods”* |  |
| Feedback in Cafeteria |  |  |  |
|  |  | *“From the beginning people were a little bit surprised with the amount of calories actually in the food, which is a bit of an eye opener, which is good”* |  |
|  |  |  |  |
|  |  | *“There were people going ‘oh there's calories on here, oh I didn’t know that’, ‘that’s surprising’”* |  |
|  |  |  |  |
|  | Positive Impact (3 of 3 sites) | *“The actual concept itself has worked very well, the fact that people do want more information I think it shows”* |  |
|  |  |  |  |
|  |  | *“I actually went for a different type of food because of the amount of calories that was in the ones that I actually wanted to go for”* |  |
|  |  |  |  |
|  |  | *“It went very well and the response that we've had is overwhelming (that) it’s gone very well and was a real success which is brilliant”* |  |
|  |  |  |  |
|  | Indifference to Information (3 of 3 sites) | *“If somebody wants a cake they're going to have a cake because they fancy cake”* |  |
|  |  |  |  |
|  |  | *“I have had comments that they don’t really care about it”* |  |
|  |  |  |  |
|  |  | *“ I think there's times where you think ‘right I’m just going to ignore it because I really want this’”* |  |
|  |  |  |  |

*Note.* Sub-themes marked with † are recurring themes that were identified in the present study and our prior

pilot study carried out across six worksite cafeterias.

**Table S1: List of non-compliant items**

| **Site** | **Product** | **Date non-compliant** |  |
| --- | --- | --- | --- |
| **Site 1** | CalypOrange | 17.04.18 |  |
|  | CalypOrange | 18.06.18 |  |
|  | CalypApple | 18.06.18 |  |
|  | CalypApple | 17.04.18 |  |
|  | Butter/Flora | 17.04.18 |  |
|  | Philadelphia | 17.04.18 |  |
|  | Cornetto Flav | 17.04.18 |  |
|  | Cornetto | 17.04.18 |  |
|  | FreshWholeFruit | 17.04.18 |  |
|  | CRISPS | 17.04.18 |  |
|  | GFChickenSldSW | 17.04.18 |  |
|  | Muller Cornr | 17.04.18 |  |
|  | DoughnutCaramel | 17.04.18 |  |
|  | Doughnutsprinkl | 17.04.18 |  |
|  | BreadPudding | 17.04.18 |  |
|  | muffblueberry | 17.04.18 |  |
|  | mufflemonpoppy | 17.04.18 |  |
|  | muffdblchoc | 17.04.18 |  |
|  | GateauxCarrot | 17.04.18 |  |
|  | GateauxVictoria | 17.04.18 |  |
|  | TrayBrownie | 17.04.18 |  |
|  | TrayChoCarShort | 17.04.18 |  |
|  | TrayRockyRoad | 17.04.18 |  |
|  | TrayMalteser | 17.04.18 |  |
|  | TrayCranbYog | 17.04.18 |  |
|  | Coco Pops | 17.04.18 |  |
|  | BranFlakes | 17.04.18 |  |
|  | Corn Flakes | 17.04.18 |  |
|  | Crunchy Nut | 17.04.18 |  |
|  | FruitNFibre | 17.04.18 |  |
|  | Rice Crispies | 17.04.18 |  |
|  | Special K | 17.04.18 |  |
|  | Weetabix | 17.04.18 |  |
|  | Alpen Original | 17.04.18 |  |
|  | InstantPorridge | 17.04.18 |  |
|  | Jaffa cake | 17.04.18 |  |
|  | ChocChipCookie | 17.04.18 |  |
|  | Spotty Cookie | 17.04.18 |  |
|  | WT Choc Cookie | 17.04.18 |  |
|  | Ryvita | 17.04.18 |  |
|  | Flapjack | 17.04.18 |  |
|  | Preserve | 01.05.18 |  |
|  | Preserve | 02.05.18 |  |
|  | Marmite | 01.05.18 |  |
|  | Marmite | 02.05.18 |  |
|  | Nutella | 02.05.18 |  |
|  | Nutella | 01.05.18 |  |
|  | Water 40 | 11.05.18 |  |
|  | Water 40 | 14.05.18 |  |
|  | GateauxVictoria | 11.05.18 |  |
|  | GateauxVictoria | 14.05.18 |  |
|  | GateauxCarrot | 11.05.18 |  |
|  | GateauxCarrot | 14.05.18 |  |
|  | WB Diet Coke | 01.06.18 |  |
| **Site 2** | CSS CRISPS WALK CHSE ONION STD. | 16.05.18 |  |
|  | CSS CRISPS WALK PRAWN CKTAIL STD | 16.05.18 |  |
|  | CSS CRISPS WALK READY SALTED STD. | 16.05.18 |  |
|  | CSS CRISPS WALK SALT VINEGAR STD. | 16.05.18 |  |
|  | CONF WINE GUMS MAYNARDS TUBE | 29.05.18 |  |
| **Site 3** | DELI SLICED HAM | 15.05.18 |  |
|  | DELI SLICED HAM | 16.05.18 |  |
|  | DELI SLICED HAM | 17.05.18 |  |
|  | JACKET POTATO | 15.05.18 |  |
|  | JACKET POTATO | 16.05.18 |  |
|  | JACKET POTATO | 17.05.18 |  |
|  | BEEF BOLOGANISE JKT FILLING | 16.05.18 |  |
|  | VEGETABLE CASSROLE JKT FILLING | 17.05.18 |  |
|  | CHILLI HAKE | 15.05.18 |  |
|  | CHIPS | 15.05.18 |  |
|  | CHIPS | 16.05.18 |  |
|  | CHIPS | 17.05.18 |  |
|  | ROAST TURKEY | 15.05.18 |  |
|  | ROAST TURKEY BAGUETTE | 15.05.18 |  |
|  | SAUSAGE GRILL | 15.05.18 |  |
|  | SIDE SALAD | 15.05.18 |  |
|  | SIDE SALAD | 16.05.18 |  |
|  | SIDE SALAD | 17.05.18 |  |
|  | CORNISH PASTRY | 16.05.18 |  |
|  | SAUSAGE GRILL | 16.05.18 |  |
|  | SAUSAGE GRILL | 17.05.18 |  |
|  | SEASONAL VEGETABLES | 16.05.18 |  |
|  | DAILY POTATOES | 17.05.18 |  |
|  | PIRI CHICKEN | 17.05.18 |  |
|  | PORK MEATBALLS | 17.05.18 |  |
|  | SEASONAL VEGETABLES | 17.05.18 |  |
|  | SPECIALITY MEATBALL BAGUETTE | 17.05.18 |  |
|  | BREAD ROLL | 15.05.18 |  |
|  | BREAD ROLL | 16.05.18 |  |
|  | BREAD ROLL | 17.05.18 |  |
|  | BROWN BREAD | 15.05.18 |  |
|  | BROWN BREAD | 16.05.18 |  |
|  | BROWN BREAD | 17.05.18 |  |
|  | THICK WHITE BREAD SLICE | 15.05.18 |  |
|  | THICK WHITE BREAD SLICE | 16.05.18 |  |
|  | THICK WHITE BREAD SLICE | 17.05.18 |  |
|  | BAGUETTE | 15.05.18 |  |
|  | BAGUETTE | 16.05.18 |  |
|  | BAGUETTE | 17.05.18 |  |
|  | BOILED EGG | 15.05.18 |  |
|  | BOILED EGG | 16.05.18 |  |
|  | BOILED EGG | 17.05.18 |  |
|  | GRATED CHEESE PORTION | 15.05.18 |  |
|  | GRATED CHEESE PORTION | 16.05.18 |  |
|  | GRATED CHEESE PORTION | 17.05.18 |  |
|  | SALAD POT TUNA MAYONNAISE | 15.05.18 |  |
|  | SALAD POT TUNA MAYONNAISE | 17.05.18 |  |
|  | DELI SANDWICH A | 15.05.18 |  |
|  | DELI SANDWICH B | 16.05.18 |  |
|  | DELI SANDWICH B | 17.05.18 |  |
|  | DELI SANDWICH C | 15.05.18 |  |
|  | DELI SANDWICH D | 15.05.18 |  |
|  | DELI SANDWICH D | 16.05.18 |  |
|  | DELI SANDWICH D | 17.05.18 |  |
|  | DELI SANDWICH E | 15.05.18 |  |
|  | DELI SANDWICH F | 17.05.18 |  |
|  | SNACKING ESSENTIALS YOG COATED PEANUTS | 01.06.18 |  |
|  |  |  |  |
|  |  |  |  |
|  |  |  |  |
